# Vaccination with *Toxoplasma gondii* calcium-dependent protein kinase 6 and rhoptry protein 18 encapsulated in poly(lactide-co-glycolide) microspheres induces long-term protective immunity in mice

**DOI:** 10.1186/s12879-016-1496-0

**Published:** 2016-04-18

**Authors:** Nian-Zhang Zhang, Ying Xu, Meng Wang, Jia Chen, Si-Yang Huang, Qi Gao, Xing-Quan Zhu

**Affiliations:** State Key Laboratory of Veterinary Etiological Biology, Lanzhou Veterinary Research Institute, Chinese Academy of Agricultural Sciences, Lanzhou, Gansu Province 730046 PR China; Key Laboratory of Animal Epidemiology and Zoonosis, Ministry of Agriculture, National Animal Protozoa Laboratory, College of Veterinary Medicine, China Agricultural University, Beijing, 100193 PR China; College of Veterinary Medicine, South China Agricultural University, Guangzhou, Guangdong Province 510642 PR China; Jiangsu Co-innovation Center for the Prevention and Control of Important Animal Infectious Diseases and Zoonoses, Yangzhou University College of Veterinary Medicine, Yangzhou, Jiangsu Province 225009 PR China

**Keywords:** *Toxoplasma gondii*, Toxoplasmosis, Rhoptry protein 18, Calcium-dependent protein kinase 6, Protein vaccine, PLG microparticles

## Abstract

**Background:**

Toxoplasmosis is a worldwide zoonosis caused by the intracellular parasite *Toxoplasma gondii.* However, no effective vaccine is yet available. Poly(lactide-co-glycolide) polymers can reduce protein degradation and sustain the release of antigens over a long period, which could generate a long-lasting immune response in vivo. Using a mouse model of toxoplasmosis, we evaluated the protective efficacy of vaccination with two recombinant proteins, which are formulated in biodegradable polymers.

**Methods:**

Two recombinant proteins, rCDPK6 and rROP18, were encapsulated in poly(d,l-lactide-co-glycolide) (PLG), and then injected subcutaneously into Kunming mice. The mice immune responses were evaluated in terms of lympho-proliferation, cytokine expression, and antibodies. The survival of infected mice and brain cyst formation were also evaluated at 6 weeks after challenge with *T. gondii* RH strain (genotype I) or PRU strain (genotype II).

**Results:**

Both protein vaccines induced Th1-biased immune responses, with increased specific antibodies and T cells, high levels of interferon-γ and interleukin 2, and strong lymphocyte proliferative responses. The mice immunized with the various protein vaccines survived slightly longer time than the control groups (*P* > 0.05) after injection with *T. gondii* RH strain. There were fewer brain cysts in the mice in all the immunized groups than that in the control groups, and the brain cysts were significantly reduced in mice immunized with proteins + 206, rCDPK6 + PLG and rCDPK6 + rROP18 + PLG (*P* < 0.05) compared controls. Further comparison of the immune responses to the proteins adjuvanted with PLG or Montanide™ ISA 206 VG 6 weeks after the last immunization revealed that antigens encapsulated in PLG conferred greater protective immunity against challenge.

**Conclusions:**

These findings suggest that the two recombinant *T. gondii* proteins encapsulated in PLG conferred immunity to *T. gondii* for an extended period, providing the foundation for the further development of a commercial vaccine against toxoplasmosis.

## Background

As the most successful parasitic pathogen, *Toxoplasma gondii* infects a wide range of warm-blooded vertebrate intermediate hosts, including humans [1–3]. The parasite is one of the most common pathogens of humans, and nearly one third of the global population is chronically infected [3, 4]. In general, *Toxoplasma gondii* infections are asymptomatic in immunocompetent individuals, but sometimes infected individuals manifest mild flu-like symptoms [3]. Congenital toxoplasmosis can cause ocular disease, intracranial calcification, hydrocephaly, microcephaly, and psychomotor and mental retardation in infants of women who experienced primary infection with *T. gondii* during gestation [5–7]. The clinical presentation of toxoplasmosis can also be severe in immunosuppressed individuals [3]. *T. gondii* infection is frequently reported in many domesticated animals in China [8–10], and causes abortion and neonatal loss, especially in goats and sheep [3]. The reemergence of food-borne routes of *T. gondii* transmission to humans has raised public health and food safety concerns in recent years [2, 4, 11].

The development of vaccine strategy against *T. gondii* infection is a public health priority because there are no effective chemical drugs to eliminate the parasite cysts [12, 13], and only a live-attenuated vaccine is available for veterinary use, based on the S48 strain of *T. gondii* (Toxovax®), has been licensed to prevent abortion in sheep [14]. Although this licensed vaccine against the parasite is recognized as a milestone in *T. gondii* vaccine studies, the safety of live-attenuated tachyzoites for humans and food-producing animals is uncertain due to virulence reversion or possible pathogenicity in individuals with compromised immune systems [13, 15]. Therefore, the development of more effective, practical, and safe vaccines against *T. gondii* infection for use in humans and animals is necessary.

Recombinant subunit vaccines may offer an efficient, safe and alternative way to immunize humans, and have been widely used to evaluate the immunogenicity of *T. gondii* antigens [13, 16–18]. The rhoptry protein ROP18 is one of the key virulence factors in the pathogenesis of *T. gondii* infection, controlling the intracellular proliferation of the parasite, and induces a protective effect against toxoplasmosis in mice. Therefore, it is considered a promising candidate vaccine against infection by this pathogen [19–21].

The calcium-dependent protein kinases (CDPKs) are a distinct protein kinase family restricted to plants, ciliates, and apicomplexans [22, 23]. Several members of the CDPK family have been identified in *T. gondii*, which participate in regulation of some critical biological processes in the parasite, including cell invasion, egress, and division [24, 25]. In previous studies, we demonstrated that the *TgCDPK1* [26] and *TgCDPK3* genes [27] are promising candidates for the development of vaccines against *T. gondii* infection, and the TgCDPK5 protein elicited only partial protection against chronic parasitic infection [28]. However, the immunogenicity of other members in the family has not been comprehensively evaluated.

Poly(lactide-co-glycolide) (PLG) polymers are a safe delivery system for antigens that can reduce protein degradation, sustain the release of antigens over a long period [29], and generate a long-lasting immune response in vivo, as revealed in previous studies, including *T. gondii* vaccines [13].

In order to identify effective vaccine candidates against both acute and chronic infection of *T. gondii* through combination of various proteins, a novel CDPK family member in *T. gondii*, designated TgCDPK6, was mixed with TgROP18 protein and the protective efficacy of this antigenic mixture against acute and chronic toxoplasmosis was evaluated. Additionally, the proteins were encapsulated in PLG microparticles and the performance of this sustained release formulation was evaluated. The sustained release of the newly combined candidate vaccines for an extended period is a step forward in the development of a commercial vaccine against *T. gondii* infection.

## Methods

### Mice

Specific-pathogen-free female Kunming mice (6–8 weeks old) were purchased from the Center of Experimental Animals, Lanzhou University, Lanzhou, China. All the mice were handled in strict accordance with the Good Animal Practice requirements of the Animal Ethics Procedures and Guidelines of the People’s Republic of China.

### Parasites

Tissue cysts of the low-virulence PRU strain (type II) and tachyzoites of the high-virulence RH strain (type I) of *T. gondii* were propagated and harvested as described in our previous studies [27, 28], and were used for the in vivo challenge of mice. The tachyzoites obtained were also used for total RNA extraction (RNAprep Pure Tissue Kit, Sangon, China) and the preparation of soluble tachyzoite antigens, as previously described [30].

### Expression and purification of recombinant proteins in *E. coli*

The complete open reading frames of the *TgCDPK6* and *TgROP18* genes were amplified with one-step reverse transcription–PCR (RT–PCR), using two pairs of specific primers, TgROP18: 5’-CG*GGATCC*ATGTTTTCGGTACAGCGGCCA-3’ (forward) and 5’-GC*GTCGAC*TTATTCTGTGTGGAGATGTTCCTG-3’ (reverse) containing *Bam*H I and *Sal* I restriction sites (italic), respectively; TgCDPK6: 5’-CCG*GGTACC*ATGGACGATCCTGCGAACTTCCGA-3’ (forward) and 5’- CGC*GCGGCCGC*TTAGTCGTGGCGCATATACGCGAC-3’ (reverse) containing *Kpn* I and *Not* I restriction sites (italic), respectively.

The two obtained RT–PCR products were inserted into the prokaryotic expression vector pET-30a(+) via their restriction sites, forming pET-ROP18 and pET-CDPK6, respectively. *Escherichia coli* strain BL21(DE3) was transformed with pET-ROP18 and pET-CDPK6, respectively and the expression of each recombinant protein was induced with 0.6 mmol/L isopropyl β-d-1-thiogalactopyranoside (Sangon), with shaking for 8 h at 26 °C. The recombinant bacteria were sonicated on ice at 400 W/s after three freeze–thaw cycles and then centrifuged at 10,000 × *g* for 30 min at 4 °C. The rROP18 and rCDPK6 proteins were purified through an Ni^2+^ column (Novagen, USA), according to the manufacturer’s instructions. The samples were analyzed with sodium dodecyl sulfate-polyacrylamide gel electrophoresis (SDS-PAGE).

### Polyclonal antibodies (pAbs)

The purified rROP18 and rCDPK6 proteins were each diluted to 500 μg/mL with PBS. Polyclonal antibodies against each protein were raised by injecting rabbits with each protein (200 μl) three times at two-week intervals. Two weeks after the last immunization, blood samples were collected from the rabbits’ ear arteries. The antibody titers were determined with enzyme-linked immunosorbent assays (ELISAs) using horseradish peroxidase (HRP)-conjugated goat anti-mouse IgG (diluted 1:5000; Sigma, USA) as the secondary antibody. Serum from a rabbit treated with PBS was used as the negative control.

### Microparticle preparation

The rTgCDPK6 and rTgROP18 proteins were each encapsulated in 50:50 PLG (Sigma) using the water-in-oil-in-water double emulsion solvent evaporation technique, according to a previous study, with minor modifications [31]. In brief, 2 mg of each protein was dissolved in 2 ml of PBS and mixed with 2 ml of 10 % polymer in dichloromethane (Sigma) to form a water-in-oil emulsion. The emulsion was then homogenized with 7 ml of 2.5 % polyvinyl alcohol (Sigma) solution to generator a stable water-in-oil-in-water double emulsion. The microparticles were collected by centrifugation at 4000 × *g* for 30 min and washed three times with distilled water to remove any non-encapsulated protein.

### Protein encapsulation

rCDPK6 + PLG and rROP18 + PLG microparticles (50 mg) were dissolved in 500 μL of 0.1 M NaOH with 2.5 % SDS at 37 °C for 4 h to extract the encapsulated proteins [29]. The reaction was terminated by adding 500 μl of HCl (0.1 M). The mixture was then centrifuged at 12,000 × g for 10 min and each supernatant was used to assess the content of the two proteins with a UV–visible light spectrophotometer (Thermo Scientific) as the absorbance at 280 nm (A_280_) and calibrated to empty PLG microparticles. The actual ratio (w/w) of each protein encapsulated in the microparticles was determined and the encapsulation efficiency (%) was calculated with the formula: (actual protein encapsulation)/(theoretical protein encapsulation) × 100 % [29]. All measurements were made in triplicate on samples from different batches.

### Duration of protein release in vitro

The microparticles (5 mg) of rCDPK6 + PLG, rROP18 + PLG and rCDPK6 + rROP18 + PLG were each suspended in 1 ml of PBS and were shaken at 37 °C in 2 ml Eppendorf tubes for 35 days. The A_280_ values for the sampled supernatants were determined daily with a UV–vis spectrophotometer (Thermo Scientific). The supernatants were added back into tubes and shaken. At 1, 7, 14, 21, 28, and 35 days, the supernatants were collected in new tubes for a western blot analysis, detected by the protein-specific rabbit pAbs. In the SDS-PAGE analysis, the loading volumes of the protein solutions in each well were identical.

### Immunization and challenge

A total of 13 groups of mice were injected subcutaneously with 10 μg of each protein. The vaccinated mice were immunized with rCDPK6, rROP18, rCDPK6 + rROP18, rCDPK6 + Montanide™ ISA 206 VG (206), rROP18 + 206, rCDPK6 + rROP18 + 206, rCDPK6 + PLG, rROP18 + PLG, or rCDPK6 + rROP18 + PLG, and the groups were designated according to the different vaccines. Mice receiving PBS alone, PBS + 206, PBS + PLG, or non-treated with anything were used as controls.

Six weeks after the second immunization, 10 mice in each group were challenged intraperitoneally with 10^3^ tachyzoites of the *T. gondii* RH strain, and their survival was recorded daily until all the mice had died. Six mice in each group were inoculated orally with 10 PRU tissue cysts, and their brain cysts were determined 4 weeks after chronic infection was established.

### Antibody assays

At weeks 0, 2, and 8, serum samples from each group were collected from the mouse tail veins to evaluate the production of specific antibodies using indirect ELISAs. The wells of 96-well microtiter plates were coated with rCDPK6 (10 μg/mL), rROP18 (10 μg/mL), or a mixture of the two proteins (each of 10 μg/mL). The nonspecific binding sites were blocked with TBS (150 mmol/L NaCl, 10 mmol/L Tris–HCl) containing 5 % bovine serum albumin. Sera (diluted 1:50) from the immunized mice were added to the wells coated with the corresponding proteins and incubated at 37 °C for 1 h. The plates were then washed by TBST (TBS + 0.05 % Tween 20) at 5 times. Each well was then incubated with HRP-conjugated anti-mouse IgG (diluted 1:250). After added 200 μl substrate solution (80 μg of 3,3′,5,5’-tetramethylbenzidine, 30 % H_2_O_2_) was added, the reaction was stopped with 2 M H_2_SO_4_. Both positive and negative controls were included in each plate. All measurements were made in triplicate at an absorbance of 450 nm.

### Lymphocyte proliferation assay

The proliferative activity of the splenocytes from each mouse group was measured with the 3-(4,5-dimethylthiazol-2-yl)-5-(3-carboxymethoxyphenyl)-2-(4-sulfophenyl)-2H-tetrazolium (MTS) method, according to our previous study [30]. The splenocytes were cultured in triplicate at a density of 2 × 10^5^ cells per well in complete medium (Dulbecco’s modified Eagle’s medium + 10 % FCS + 100 U/ml penicillin/streptomycin). For the tests, the cells were stimulated with the corresponding antigens (A, 10 μg/ml), and cells to which medium (M) alone was added were used as the negative control. The stimulation index (SI) was calculated with the formula: SI = OD_570A_/OD_570M_.

### Flow cytometry

The percentage of CD4^+^ T cells and CD8^+^ T cells present in the spleens were determined by using flow cytometry according to our previous study [30]. The CD3, CD4, and CD8 antigens on the surfaces of the T-cell subclasses were stained with the corresponding anti-mouse antibodies (eBioscience) tagged with phycoerythrin (PE), allophycocyanin (APC), and fluorescein isothiocyanate (FITC), respectively. The fluorescence profiles of all the samples were analyzed on a FACScan flow cytometer (BD Biosciences) using the System II Software (Coulter).

### Cytokine assays

The levels of cytokines secreted by the cultured splenocytes were measured with commercial ELISA kits (BioLegend, USA), as described in previous studies [13, 30]. The analyses were performed in triplicate.

### Statistical analysis

All statistical analyses were performed in GraphPad Prism 5.0 or SAS (Statistical Analysis System, version 8.0). The differences in antibody responses, lympho-proliferation assays, cytokine production, and percentages of CD4^+^ and CD8^+^ T cells were compared with one-way ANOVA. Differences in the comparisons of groups were considered significant at *P* < 0.05.

## Results

### Expression of rCDPK6 and rROP18 proteins

*Escherichia coli* BL21(DE3) cells were transformed with either the prokaryotic expression vector pET-CDPK6 or pET-ROP18. Each recombinant protein was separated with SDS-PAGE and visualized with Coomassie Brilliant Blue staining.

### Protein encapsulation rate and release rate from microparticles in vitro

The encapsulated proteins were extracted with NaOH. After centrifugation, the supernatants were analyzed with a UV–Vis spectrophotometer (Thermo Scientific). The encapsulation efficiency for the rCDPK6 protein ranged from 57.3 % to 67.7 % and that for the rROP18 protein ranged from 58.1 % to 72.3 %. There were no significant differences (*P* > 0.05) among the three batches of each type of microparticle.

**Fig. 1:**
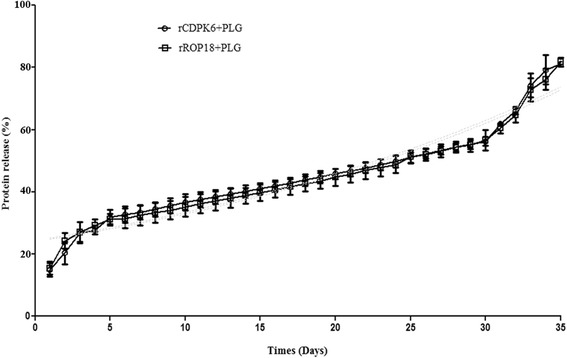
Detection of rCDPK6 and rROP18 proteins release from PLGs in vitro

The concentrations of the rCDPK6 and rROP18 proteins released from each type of microparticle were tested daily until day 35. The rCDPK6 and rROP18 proteins were released gradually from the microparticles. At day 5, about 70 % of each protein was still loaded in the microparticles, and nearly 30 % of each protein was rapidly released in week 5. Altogether, more than 80 % of each protein was released over the 35 days of the study (Fig. 1).

The antigenicity of the released rCDPK6 and rROP18 proteins collected on days 1, 7, 14, 21, 28, and 35 were determined by western blotting. The released proteins were identified by their corresponding specific Abs, suggesting the potential capacity of the PLG-encapsulated antigens to elicit protective immunity against *T. gondii* infection in the long term.

### Antibody responses

**Fig. 2:**
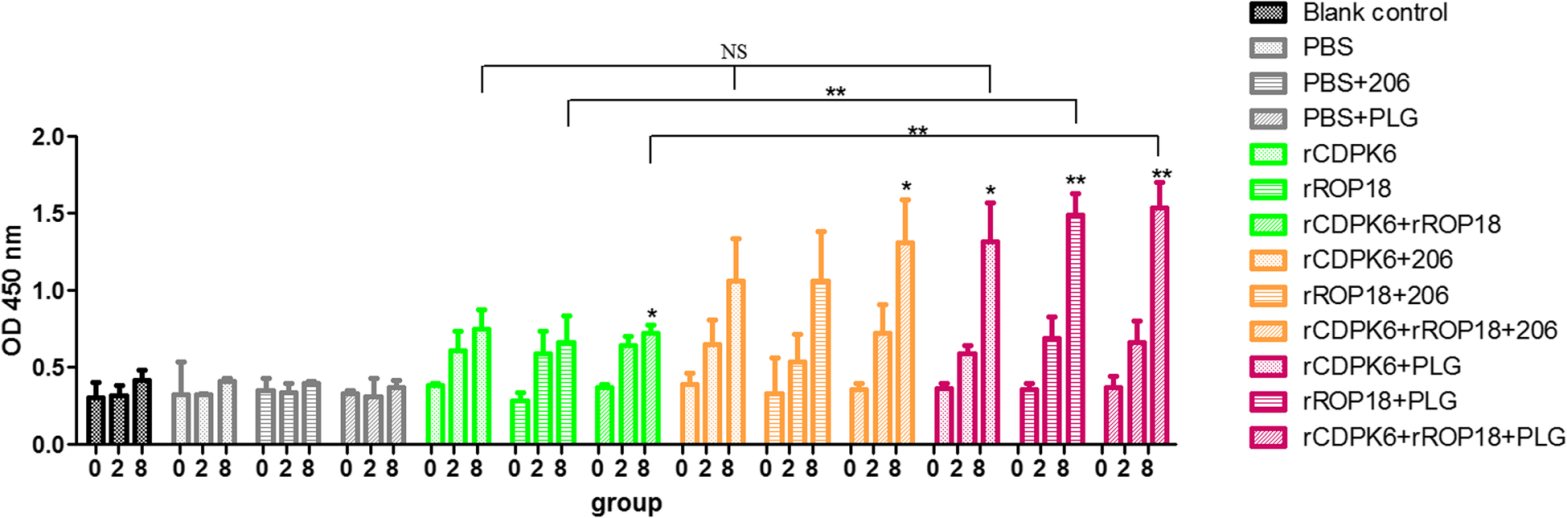
IgG antibodies induced by various protein vaccines in the sera of mice at 0, 2, and 8 weeks. Each bar represents the mean OD (± SE, *n* = 3). *P < 0.05, **P < 0.01 compared with the controls. NS: not significant

Three serum samples from each vaccinated mouse group were collected at 0, 2, and 8 weeks to detect specific IgG antibodies. All the protein vaccines elicited specific humoral responses and the levels of IgG antibodies in the experimental mice increased sequentially with successive immunizations with the tested antigens, and reached their highest levels in week 8 (Fig. 2). However, no significant differences in the antibody levels were detected between pre- and post-immunization in the control groups. Six weeks after the last vaccination, the levels of IgG antibodies in the mice immunized with rROP18 + PLG or rCDPK6 + rROP18 + PLG were significantly higher than those in the mice immunized with rROP18 or rCDPK6 + rROP18 (*P* < 0.01), but were not statistically different from those in the mice immunized with various proteins + 206 adjuvant (*P* > 0.05) (Fig. 2).

### Evaluation of splenocyte proliferation

**Fig. 3:**
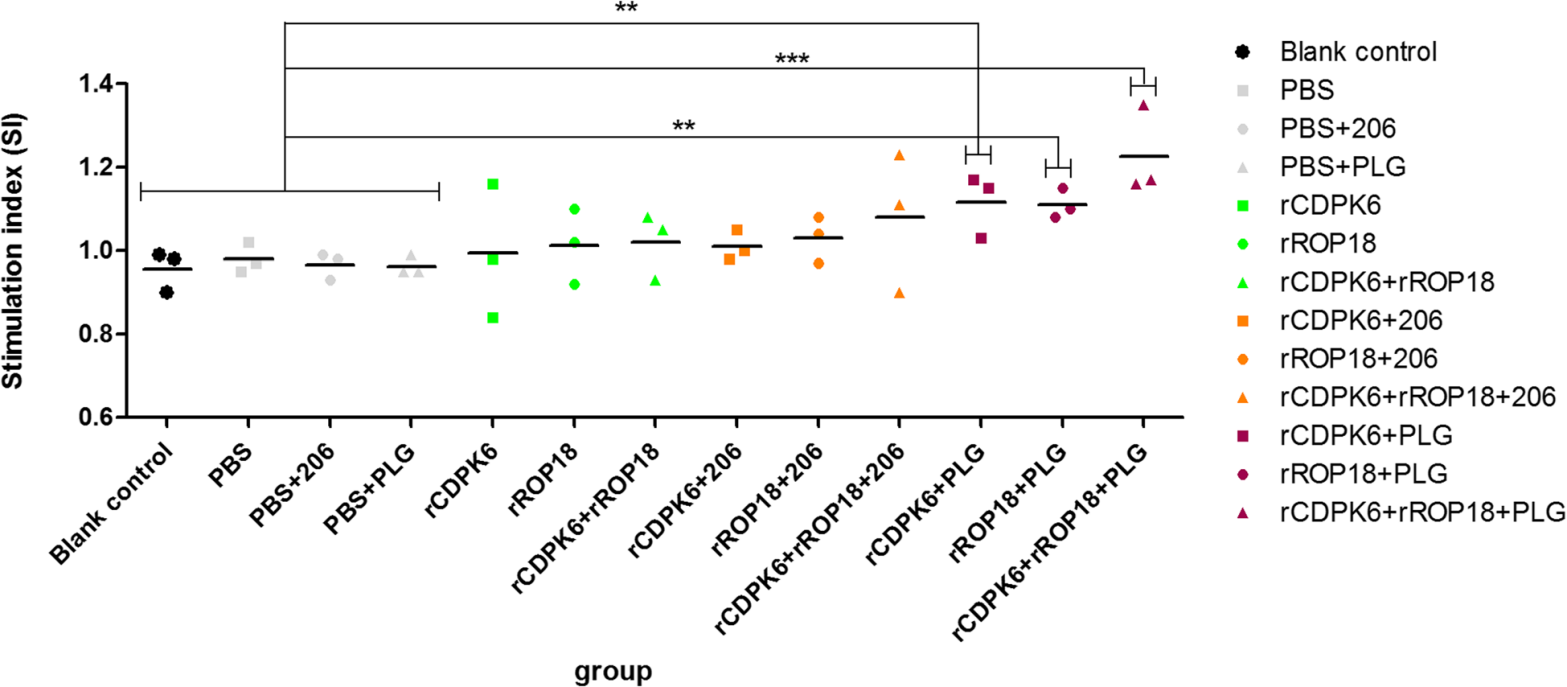
Splenocyte proliferative responses in immunized mice. *P < 0.05. ** *P* < 0.01

The proliferative responses of splenocytes from all mouse groups were tested with ELISAs after they had been co-cultured with MTS for 96 h. The splenocytes proliferated more strongly in the vaccinated mice than in the controls (Fig. 3), and the highest proliferative response was detected in the mice immunized with rCDPK6 + rROP18 + PLG (*P* < 0.001). The proliferation SIs of the four control groups did not differ significantly (*P* > 0.05).

### Percentages of CD4^+^ and CD8^+^ T lymphocytes

**Fig. 4:**
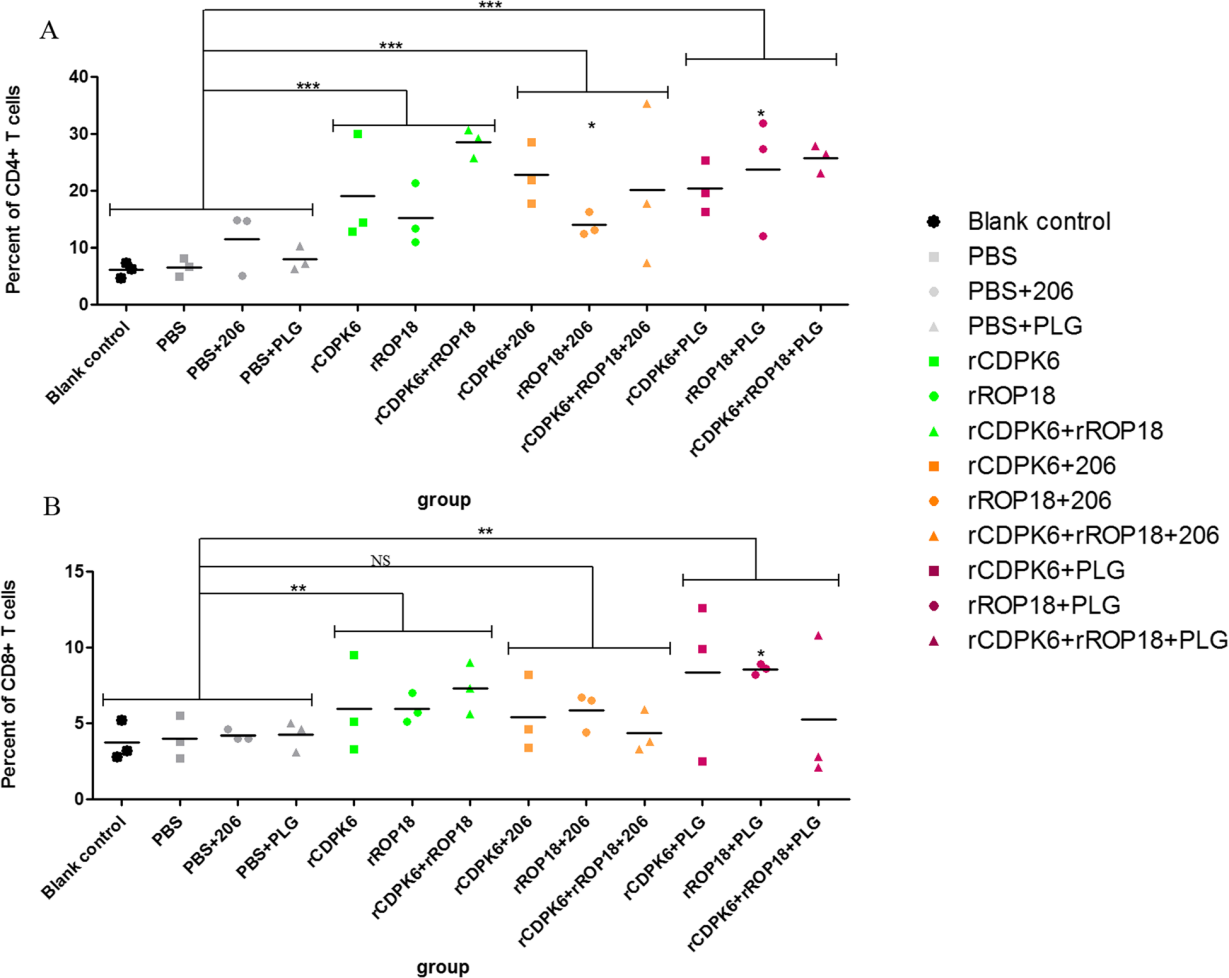
Percentages of T-cell subsets (**a** CD4+; **b** CD8+ T cells) in immunized mice. The 4 control groups, PBS + PLG, PBS + 206, PBS and blank control, were treated as controls in statistical analysis. The *P* value was calculated using the measurements from each immunized group to compare with the average from controls. Furthermore, the proteins + PLG, proteins + 206 and proteins alone were respectively treated as a group compared with the average from controls. **P < 0.01. *** *P* < 0.001. NS: not significant

The percentages of CD4^+^ and CD8^+^ T cells in the total splenocytes in each group were determined with flow cytometry, and the results are shown in Fig. 4. Mice immunized with the various protein vaccines generated higher levels of both T-cell subtypes than those in the controls. Immunization of mice with protein–PLG microparticles induced significantly higher levels of CD4^+^ (*P* < 0.001) and CD8^+^ T lymphocytes (*P* < 0.01) than did the controls. The percentages of CD4^+^ cells in mice immunized with rROP18 + 206 and rROP18 + PLG were significantly higher than that in controls (*P* < 0.05). Only mice from rROP18 + 206 group were showed significantly higher level of CD8^+^ T cells compared to that in controls (*P* < 0.05).

### Cytokine production by spleen cells

The effects of immunization on cytokine production in vitro after stimulation with specific antigens were investigated with ELISAs. As shown in Table 1, the immunization of mice with various proteins significantly increased the production of interferon-γ (IFN-γ; *P* < 0.05) and interleukin-2 (IL-2; *P* < 0.05). In contrast, the levels of cytokines IL-4 and IL-10 were significantly reduced compared with those in the control groups (*P* < 0.05). The levels of IL-12 in mice vaccinated with each protein did not differ significantly from those in the controls (*P* > 0.05).

**Table 1:** Cytokine production by splenocytes of immunized Kunming mice after stimulation with toxoplasma lysate antigen^a^

Group	Cytokine production (pg/ml)				
	IFN-γ	IL-2	IL-4	IL-10	IL-12 (p70)
CDPK6 + ROP18 + PLG	2092.66 ± 363.69***	238.48 ± 6.37***	>0*	360.61 ± 296.21	4.55 ± 2.91
CDPK6 + ROP18 + 206	1727.43 ± 506.06**	257.92 ± 4.04***	>0*	520.98 ± 91.89***	<0
CDPK6 + ROP18	1835.37 ± 384.89**	251.35 ± 5.51***	>0*	409.57 ± 337.62	<0
ROP18 + PLG	1720.61 ± 329.18**	243.76 ± 91.02**	>0*	56.92 ± 57.09***	9.17 ± 15.2
ROP18 + 206	1595.37 ± 1204.87	263.7 ± 30.02***	>0*	430.08 ± 623.13	<0
ROP18	1530.8 ± 724.92*	244.52 ± 1.82***	>0*	484.58 ± 453.58	<0
CDPK6 + PLG	1040.08 ± 477.05	179.65 ± 107.16	>0*	139.41 ± 141.62	>0
CDPK6 + 206	1013.52 ± 630.91	215.95 ± 43.12***	>0*	121.55 ± 103.54	<0
CDPK6	838.58 ± 186.37	85.66 ± 140.77	>0*	>0	<0
PBS + PLG	471.15 ± 232.97	45.11 ± 46.95	9.29 ± 3.56	609.99 ± 267.23	6.12 ± 7.25
PBS + 206	611.01 ± 308.16	38.89 ± 35.46	13.09 ± 4.67	613.01 ± 154.41	1.09 ± 2.26
PBS	515.27 ± 363.03	20.76 ± 32.26	14.73 ± 2.46	597.48 ± 216.81	<0
Control	590.3 ± 257.92	33.9 ± 13.16	12.43 ± 12.92	606.35 ± 376.32	<0

^a^ Splenocytes from mice were harvested 6 weeks after the last immunization

The 4 control groups, PBS + PLG, PBS + 206, PBS, and blank control, were treated as controls in regard to statistical analysis. *P < 0.05. **P < 0.01. ***P < 0.001 compared with the average of the controls

### Protective activity of vaccination with protein antigens

**Fig. 5:**
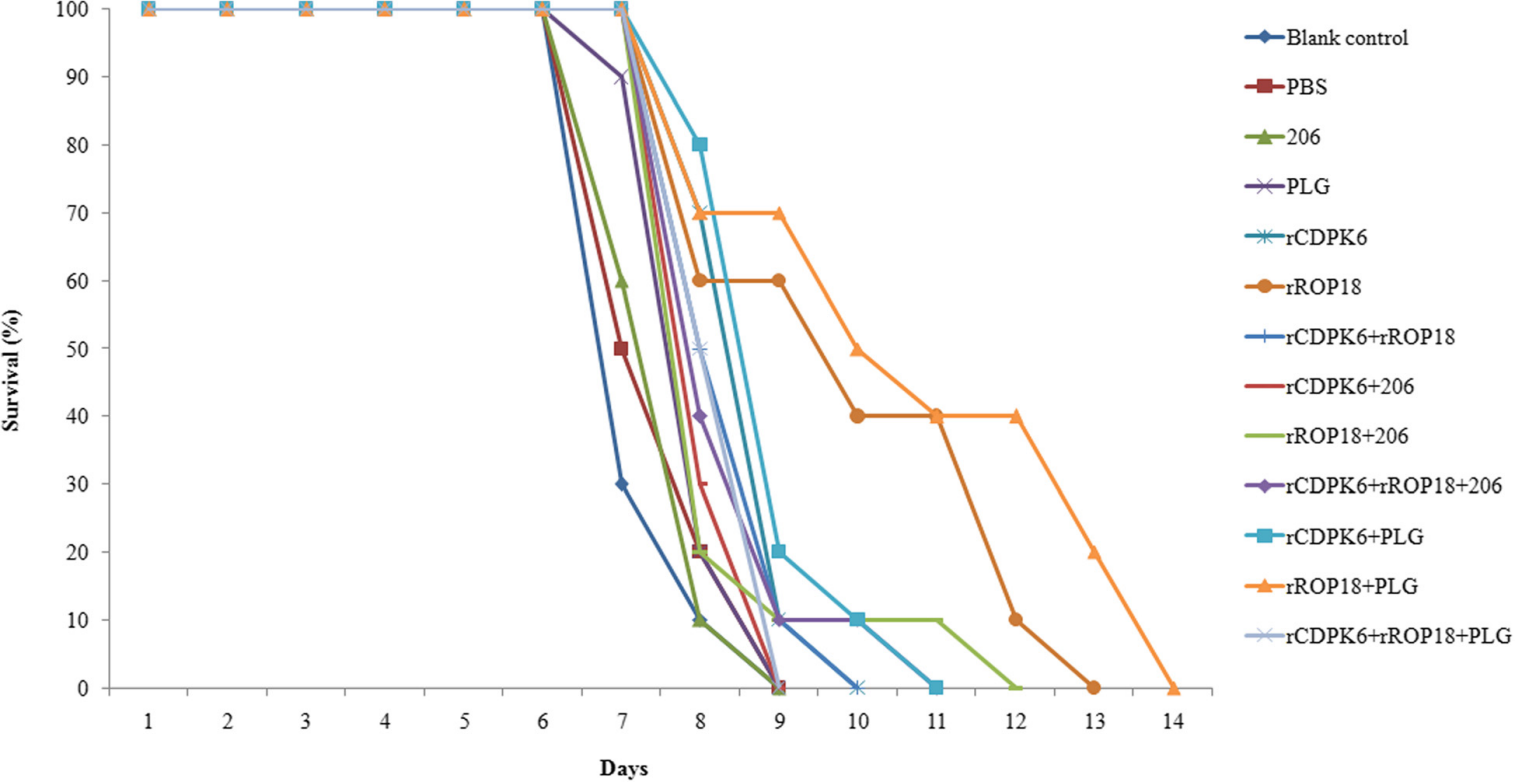
Survival rates of mice immunized with various protein vaccines after challenge with *Toxoplasma gondii*. Kunming mice were challenged with 1 × 10^3^ tachyzoites of *Toxoplasma gondii* RH strain, 6 weeks after the second immunization

At 6 weeks after vaccination, 10 mice from each group were challenged with 10^3^ tachyzoites of the *T. gondii* RH strain and 10 cysts of the PRU strain. The average survival time of the mice immunized with the various protein vaccines (8.56 days) was slightly longer than that in the controls (8 days) (Fig. 5). Immunization with rROP18 + PLG (10.9 days ± 2.58), rROP18 (10.1 days ± 1.52), and rCDPK6 + PLG (9.1 days ± 0.24) was showed to significantly prolong the average survival time in mice compared with that of the controls after challenged with RH strain (*P* < 0.05).

**Fig. 6:**
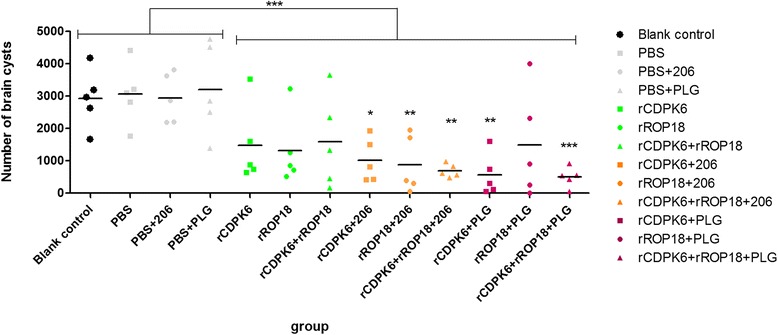
Number of tissue cysts per brain after challenge with *Toxoplasma gondii* cysts in mice from all groups. Mice immunized with various protein vaccines and in controls were challenged with 10 tissue cysts of *T. gondii* PRU strain, 6 weeks after the last booster. The 4 control groups, PBS + PLG, PBS + 206, PBS and blank control, were treated as controls in statistical analysis. The P value was calculated using the measurements from each immunized group to compare with the average from controls. Furthermore, the numbers of brain cysts in mice immunized with various proteins were compared with that in controls. ***P < 0.001

As shown in Figure 6, immunization with the different proteins partially protected the mice against chronic *T. gondii* infection. The tissue cyst loadings in the brains of mice vaccinated with the various proteins varied from 47.7 % to 73.6 %, and were significantly lower than those in the control groups (*P* < 0.001). The protein antigens plus PLG microparticles provided more effective protection to the mice than was observed in the corresponding groups of mice immunized only with proteins. However, the differences were not statistically significant (*P* > 0.05).

## Discussion

In this study, vaccination with the rROP18 or rCDPK6 protein induced specific humoral and cellular immune responses. Infected vaccinated mice showed slightly improved survival time and reduced brain cyst burden. These results suggest that these two recombinant *T. gondii* antigens are attractive targets for further validation and that PLG maintains the immunogenicity of the encapsulated proteins.

Recombinant protein vaccines have been used in numerous immunological studies in veterinary medicine in recent years [13] because they can induce systemic humoral and cellular immune responses [32–35] and are amenable to large-scale production. However, recombinant proteins can be easily proteolytically degraded in the vaccinated animals, which necessitate more frequent immunizations. Microparticles of PLG, a biodegradable and biocompatible polymer, can protect proteins from degradation for long periods [36]. The sustained release of these antigens should facilitate their uptake by antigen-presenting cells, which would elicit a strong immune response [37]. When PLG was used as a delivery system to evaluate the protective efficacy of anti-*Toxoplasma* vaccines, significantly long-lasting immune responses and effective protection against *T. gondii* infection were induced in vaccinated mice [38, 39]. Here, we immunized mice with protein vaccines twice, and the proteins encapsulated in PLG microspheres elicited stronger humoral and cellular immune responses compared to the control mice. Also, vaccination using encapsulated proteins maintained the protective activity for 6 weeks after the last immunization. This suggests that PLG may be an attractive alternative to develop long-lasting vaccines against *T. gondii* infection in humans and animals.

*Toxoplasma gondii* ROP18 protein is considered a promising candidate target for vaccination against acute toxoplasmosis in mice [16, 19], and a specific CD8^+^ T-cell response against TgROP18-derived peptides has also been observed in patients with chronic asymptomatic toxoplasmosis, suggesting the potential immunogenicity of TgROP18 in patients with chronic toxoplasmosis [40]. Our previous study predicted 10 potential epitopes distributed on TgCDPK6, indicating that it is a potential vaccine antigen against *T. gondii* [41]. Here, mice immunized with rCDPK6 produced high levels of IgG antibodies and a strong lymphoproliferative response compared with those of the controls. In a chronic infection model, the brain cyst burden in rCDPK6-immunized mice was reduced by more than 40 % compared to the level in the controls. Together with the results for TgROP18, these findings suggested that these two proteins (TgROP18 and TgCDPK6) may be used in recombinant multi-antigen vaccines against *T. gondii* infection*.*

Th1 immunity has been shown to play a critical role in the host’s defenses against intracellular pathogens, primarily via an IFN-γ-driven cytotoxic T-cell (CTL) response [42]. IFN-γ, produced by natural killer (NK) cells and T cells, plays a crucial role in host survival because it promotes multiple intracellular mechanisms that constrain the replication of the parasite (e.g., nitric oxide) [43–47] or even kill *T. gondii*. IFN-γ-deficient mice showed extreme susceptibility to infection with the parasite [48]. IFN-γ combined with IL-2 is also involved in preventing parasitic invasion [49]. Another characteristic cytokine of Th1 immunity is IL-12, an innate product of the host’s Toll-like receptor/MyD88 response after *T. gondii* infection [50]. IL-12 is an important effector, and only mediates the adaptive immune response, thus participating in the regulation of IFN-γ [51]. All the proteins investigated in the present study induced a significant increase in cytokines INF-γ and IL-2, which are essential for the prolonged survival and reduced brain cysts in the immunized mice. The mixed-antigen vaccine produced higher levels of INF-γ and IL-2 than either protein alone. In contrast, IL-4 and IL-10, Th2-based cytokines, were reduced in the immunized mice. These results suggest that the cellular immune responses induced by the two protein antigens were primarily skewed to a Th1-predominant immune response.

CD8^+^ T cells are specialized to recognize and destroy infected host cells by the production of IFN-γ or perforin-mediated cytolysis [52, 53], which are also critical in mediating the resistance to *T. gondii* infection. CD8^+^ T cells that mediate this protection should combine with CD4^+^ T cells to play a synergistic role [53, 54]. In the protein-immunized mice, both T-cell subsets were increased compared with those in the controls. This tendency is consistent with the results of Th1-type cytokines, especially IFN-γ, and is consistent with the mechanisms underlying the protective effects of these vaccines.

The IgG antibodies specifically directed against *T. gondii* opsonize the parasite for phagocytosis, block invasion, and are also related to the activation of the classical complement pathway in those who are acutely susceptible to toxoplasmosis [55, 56]. Mice vaccinated with the proteins in this study displayed higher specific IgG antibody titers than the controls, and the IgG antibody titers were especially high in the group immunized with rCDPK6 + rROP18 plus PLG, which may have contributed to the significant reduction in tissue cyst burden in the brains of this group.

Immunization with a recombinant multi-antigen vaccine showed a slightly increased average survival (8.56 days) compared the controls, which died within 8 days of challenge with *T. gondii* (*P* > 0.05). TgROP18 and TgCDPK6 have the advantage of protecting against acute and chronic toxoplasmosis, respectively, but when the two recombinant proteins were mixed, the effect against acute *T. gondii* infection showed no synergistic effect, contrary to our expectation. In addition to increasing the numbers of CTL epitopes and detecting new *T. gondii* antigens, as we noted previously [13], the immunization strategies and the ratio of each antigen in the mixed protein vaccine should be evaluated in future studies.

## Conclusions

Our study demonstrated that vaccination of mice with rCDPK6 can elicit Th1-biased responses against acute and chronic *T. gondii* infections. However, a combination with rROP18 did not enhance the immune responses or provide better protective efficacy against *T. gondii* infection compared to each single protein. Encapsulation of proteins in PLG maintains the immunogenicity for a longer period. Evaluation of the potential of PLG in the development of a vaccine against toxoplasmosis should be further studied.

### Ethics approval and consent to participate

This study was approved by the Animal Ethics Committee of Lanzhou Veterinary Research Institute, Chinese Academy of Agricultural Sciences (Approval No. LVRIAEC2013-016).

### Consent for publication

Not applicable.

### Availability of data and materials

All the data supporting our findings is contained within the manuscript.

## Abbreviations

206: Montanide™ ISA 206 VG
APC: allophycocyanin
CDPK: calcium-dependent protein kinase
CTL: cytotoxic T-cell
ELISA: enzyme-linked immunosorbent assay
FITC: fluorescein isothiocyanate
HRP: horseradish peroxidase
IFN-γ: interferon-γ
IL-2: interleukin-2
MTS: 3-(4,5-dimethylthiazol-2-yl)-5-(3-carboxymethoxyphenyl)-2-(4-sulfophenyl)-2H-tetrazolium
MyD88: myeloid differentiation factor 88
NK cells: natural killer cells
PBS: phosphate buffer solution
PE: phycoerythrin
PLG: poly(d,l-lactide-co-glycolide)
ROP: rhoptry protein
RT–PCR: reverse transcription–polymerase chain reaction
SAS: statistical analysis system
SDS-PAGE: sodium dodecyl sulfate-polyacrylamide gel electrophoresis
SI: stimulation index
TBST: tris buffered saline with Tween 20

## References

[1] Robert-Gangneux F, Darde ML (2012). Epidemiology of and diagnostic strategies for toxoplasmosis. Clin Microbiol Rev.

[2] Cenci-Goga BT, Rossitto PV, Sechi P, McCrindle CM, Cullor JS (2011). *Toxoplasma* in animals, food, and humans: an old parasite of new concern. Foodborne Pathog Dis.

[3] Dubey JP (2010). Toxoplasmosis of animals and humans.

[4] Zhou P, Chen ZG, Li HL, Zheng HH, He SY, Lin RQ, Zhu XQ (2011). *Toxoplasma gondii* infection in humans in China. Parasit Vectors.

[5] Gebremedhin EZ, Abebe AH, Tessema TS, Tullu KD, Medhin G, Vitale M, Di Marco V, Cox E, Dorny P (2013). Seroepidemiology of *Toxoplasma gondii* infection in women of child-bearing age in central Ethiopia. BMC Infect Dis.

[6] Kieffer F, Wallon M, Garcia P, Thulliez P, Peyron F, Franck J (2008). Risk factors for retinochoroiditis during the first 2 years of life in infants with treated congenital toxoplasmosis. Pediatr Infect Dis J.

[7] Gras L, Wallon M, Pollak A, Cortina-Boria M, Evenqard B, Hayde M, Petersen E, Gilbert R (2005). European muliticenter study on congential toxoplasmosis: association between prenatal treatment and clinical manifestations of congenital toxoplasmosis in infancy: a cohort study in 13 European centres. Acta Paediatr.

[8] Jiang HH, Huang SY, Zhou DH, Zhang XX, Su CL, Deng SZ, Zhu XQ (2013). Genetic characterization of *Toxoplasma gondii* from pigs from different localities in China by PCR-RFLP. Parasit Vectors.

[9] Wang M, Wang YH, Ye Q, Meng P, Yin H, Zhang DL (2012). Serological survey of *Toxoplasma gondii* in Tibetan mastiffs (*Canis lupus familiaris*) and yaks (*Bos grunniens*) in Qinghai, China. Parasites Vectors.

[10] Zhou P, Chen N, Zhang RL, Lin RQ, Zhu XQ (2008). Food-borne parasitic zoonoses in China: perspective for control. Trends Parasitol.

[11] Elmore SA, Jones JL, Conrad PA, Patton S, Lindsay DS, Dubey JP (2010). *Toxoplasma gondii*: epidemiology, feline clinical aspects, and prevention. Trends Parasitol.

[12] Innes EA (2010). Vaccination against *Toxoplasma gondii*: an increasing priority for collaborative research?. Expert Rev Vaccines.

[13] Zhang NZ, Chen J, Wang M, Petersen E, Zhu XQ (2013). Vaccines against *Toxoplasma gondii*: new developments and perspectives. Expert Rev Vaccines.

[14] Buxton D, Innes EA (1995). A commercial vaccine for ovine toxoplasmosis. Parasitology.

[15] Jongert E, Roberts CW, Gargano N, Forster-Waldl E, Petersen E (2009). Vaccines against *Toxoplasma gondii*: challenges and opportunities. Mem Inst Oswaldo Cruz.

[16] Qu DF, Han JZ, Du AF (2013). Enhancement of protective immune response to recombinant *Toxoplasma gondii* ROP18 antigen by ginsenoside Re. Exp Parasitol.

[17] Huang X, Li J, Zhang G, Gong P, Yang J, Zhang X (2012). *Toxoplasma gondii*: protective immunity against toxoplasmosis with recombinant actin depolymerizing factor protein in BLAB/c mice. Exp Parasitol.

[18] Tan F, Hu X, Luo FJ, Pan CW, Chen XG (2011). Induction of protective Th1 immune responses in mice by vaccination with recombinant *Toxoplasma gondii* nucleoside triphosphate hydrolase-II. Vaccine.

[19] Yuan ZG, Zhang XX, Lin RQ, Petersen E, He S, Yu M, He XH, Zhou DH, He Y, Li HX, Liao M, Zhu XQ (2011). Protective effect against toxoplasmosis in mice induced by DNA immunization with gene encoding *Toxoplasma gondii* ROP18. Vaccine.

[20] EI Hajj H, Lebrun M, Arold ST, Vial H, Labesse G, Dubremetz JF (2007). ROP18 is a rhoptry kinase controlling the intracellular proliferation of *Toxoplasma gondii*. PLoS Pathog.

[21] Saeij JP, Boyle JP, Coller S, Taylor S, Sibley LD, Brooke-Powell ET, Ajioka JW, Boothroyd JC (2006). Polymorphic secreted kinases are virulence factors in toxoplasmosis. Science.

[22] Billker O, Lourido S, Sibley LD (2009). Calcium-dependent signaling and kinases in apicomplexan parasites. Cell Host Microbe.

[23] Nagamune K, Sibley LD (2006). Comparative genomic and phylogenetic analyses of calcium ATPases and calcium-regulated proteins in the apicomplexa. Mol Biol Evol.

[24] Lourido S, Shuman J, Zhang C, Shokat KM, Hui R, Sibley LD (2013). Calcium-dependent protein kinase 1 is an essential regulator of exocytosis in *Toxoplasma*. Nature.

[25] McCoy JM, Whitehead L, van Dooren GG, Tonkin CJ (2012). TgCDPK3 regulates calcium-dependent egress of *Toxoplasma gondii* from host cells. PLoS Pathog.

[26] Chen J, Li ZY, Huang SY, Petersen E, Song HQ, Zhou DH, Zhu XQ (2014). Protective efficacy of *Toxoplasma gondii* calcium-dependent protein kinase 1 (TgCDPK1) adjuvated with recombinant IL-15 and IL-21 against experimental toxoplasmosis in mice. BMC Infect Dis.

[27] Zhang NZ, Huang SY, Zhou DH, Chen J, Xu Y, Tian WP, Lu J, Zhu XQ (2013). Protective immunity against *Toxoplasma gondii* induced by DNA immunization with the gene encoding a novel vaccine candidate: calcium-dependent protein kinase 3. BMC Infect Dis.

[28] Zhang NZ, Huang SY, Xu Y, Chen J, Wang JL, Tian WP, Zhu XQ (2014). Evaluation of immune responses in mice after DNA immunization with putative *Toxopalsma gondii* calcium-dependent protein kinase 5. Clin Vaccine Immunol.

[29] Jeffery H, Davis SS, O’Hagan DT (1993). The preparation and characterization of poly (lactide-co-glycolide) microparticles. II. The entrapment of a model protein using a (water-in-oil)-in-water emulsion solvent evaporation technique. Pharm Res.

[30] Chen J, Huang SY, Li ZY, Yuan ZG, Zhou D, Petersen E, Zhang NZ, Zhu XQ (2013). Protective immunity induced by a DNA vaccine expressing eIF4A of *Toxoplasma gondii* against acute toxoplasmosis in mice. Vaccine.

[31] Acharya AP, Lewis JS, Keselowsky BG (2013). Combinatorial co-encapsulation of hydrophobic molecules in poly (lactide-co-glycolide) microparticles. Biomaterials.

[32] Letscher-Bru V, Villard O, Risse B, Zauke M, Klein JP, Kien TT (1998). Protective effect of vaccination with a combination of recombinant surface antigen 1 and interleukin-12 against toxoplasmosis in mice. Infect Immun.

[33] Golkar M, Shokrgozar MA, Rafati S, Musset K, Assmar M, Sadaie R, CesbronDelauw MF, Mercier C (2007). Evaluation of protective effect of recombinant dense granule antigens GRA2 and GRA6 formulated in monophosphoryl lipid A (MPL) adjuvant against *Toxoplasma* chronic infection in mice. Vaccine.

[34] Cuppari AF, Sanchez V, Ledesma B, Frank FM, Goldman A, Angel SO, Martin V (2008). *Toxoplasma gondii* protease inhibitor-1 (TgPI-1) is a novel vaccine candidate against toxoplasmosis. Vaccine.

[35] Dziadek B, Gatkowska J, Brzostek A, Dziadek J, Dzitko K, Dlugonska H (2009). *Toxoplasma gondii*: the immunogenic and protective efficacy of recombinant ROP2 and ROP4 rhoptry proteins in murine experimental toxoplasmosis. Exp Parasitol.

[36] Sinha VR, Trehan A (2003). Biodegradable microspheres for protein delivery. J Control Release.

[37] Lim TY, Poh CK, Wang W (2009). Poly (lactic-co-glycolic acid) as a controlled release delivery device. J Mater Sci Mater Med.

[38] Chuang SC, Ko JC, Chen CP, Du JT, Yang CD (2013). Encapsulation of chimeric protein rSAG1/2 into poly (lactide-co-glycolide) microparticles induces long-term protective immunity against *Toxoplasma gondii* in mice. Exp Parasitol.

[39] Chuang SC, Ko JC, Chen CP, Du JT, Yang CD (2013). Induction of long-lasting protective immunity against *Toxoplasma gondii* in BALB/c mice by recombinant surface antigen 1 protein encapsulated in poly (lactide-co-glycolide) microparticles. Parasit Vectors.

[40] Torres-Morales E, Taborda L, Cardona N, De-la-Torre A, Sepulveda-Arias JC, Patarroyo MA, Gomez-Marin JE (2014). Th1 and Th2 immune response to P30 and ROP18 peptides in human toxoplasmosis. Med Microbiol Immunol.

[41] Zhang NZ, Huang SY, Zhou DH, Xu Y, He JJ, Zhu XQ (2014). Identification and bioinformatics analysis of a putative calcium-dependent protein kinase (CDPK6) from *Toxoplasma gondii*. Genet Mol Res.

[42] Sibley LD (2011). Invasion and intracellular survival by protozoan parasites. Immunol Rev.

[43] Nathan CF, Murray HW, Wiebe ME, Rubin BY (1983). Identification of interferon-gamma as the lymphokine that activates human macrophage oxidative metabolism and antimicrobial activity. J Exp Med.

[44] Suzuki Y, Orellana MA, Schreiber RD, Remington JS (1988). Interferon-gamma: the major mediator of resistance against *Toxoplasma gondii*. Science.

[45] Scharton-Kersten TM, Wynn TA, Denkers EY, Bala S, Grunvald E, Hieny S, Gazzinelli RT, Sher A (1996). In the absence of endogenous IFN-gamma, mice develop unimpaired IL-12 responses to *Toxoplasma gondii* while failing to control acute infection. J Immunol.

[46] Bessieres MH, Swierczynski B, Cassaing S, Miedouge M, Olle P, Sequela JP, Pipy B (1997). Role of IFN-gamma, TNF-alpha, IL-4 and IL-10 in the regulation of experimental *Toxoplasma gondii* infection. J Eukaryot Microbiol.

[47] Sturge CR, Benson A, Raetz M, Wilhelm CL, Mirpuri J, Vitetta ES, Yarovinsky F (2013). TLR-independent neutrophil-derived IFN-γ is important for host resistance to intracellular pathogens. Proc Natl Acad Sci U S A.

[48] Yap GS, Sher A (1999). Effector cells of both nonhemopoietic and hemopoietic origin are required for interferon (IFN)-gamma- and tumor necrosis factor (TNF)-alpha-dependent host resistance to the intracellular pathogen, *Toxoplasma gondii*. J Exp Med.

[49] Matowicka-Karna J, Dymicka-Piekarska V, Kemona H (2009). Does *Toxoplasma gondii* infection affect the levels of IgE and cytokines (IL-5, IL-6, IL-10, IL-12, and TNF-alpha)?. Clin Dev Immunol.

[50] LaRosa DF, Stumhofer JS, Gelman AE, Rahman AH, Taylor DK, Hunter CA, Turka LA (2008). T cell expression of MyD88 is required for resistance to *Toxoplasma gondii*. Proc Natl Acad Sci U S A.

[51] Khan IA, Matsuura T, Kasper LH (1994). Interleukin-12 enhances murine survival against acute toxoplasmosis. Infect Immun.

[52] Denkers EY, Yap G, Scharton-Kersten T, Charest H, Butcher BA, Caspar P, Heiny S, Sher A (1997). Perforin-mediated cytolysis plays a limited role in host resistance to *Toxoplasma gondii*. J Immunol.

[53] Gazzinelli R, Xu Y, Hieny S, Cheever A, Sher A (1992). Simultaneous depletion of CD4+ and CD8+ T lymphocytes is required to reactivate chronic infection with *Toxoplasma gondii*. J Immunol.

[54] Kimberly AJ, Christopher AH (2010). Regulation of CD8+ T cell responses to infection with parasitic protozoa. Exp Parasitol.

[55] Sayles PC, Gibson GW, Johnson LL (2000). B cells are essential for vaccination- induced resistance to virulent *Toxoplasma gondii*. Infect Immun.

[56] Vercammen M, Scorza T, El Bouhdidi A, Van Beeck K, Carlier Y, Dubremetz JF, Verschueren H (1999). Opsonization of *Toxoplasma gondii* tachyzoites with nonspecific immunoglobulins promotes their phagocytosis by macrophages and inhibits their proliferation in nonphagocytic cells in tissue culture. Parasite Immunol.

